# Fetal Growth Restriction: Contemporary Evidence to Guide Delivery Timing and Intrapartum Management

**DOI:** 10.3390/diagnostics16050806

**Published:** 2026-03-09

**Authors:** Ana Carolina Rabachini Caetano, Ana Cristina Perez Zamarian, Luciano Marcondes Machado Nardozza, Seizo Miyadahira, Giselle Darahem Tedesco, Lara Dariolli Rossi, Gustavo Yano Callado, Edward Araujo Júnior, Alessandra Cristina Marcolin

**Affiliations:** 1 Department of Obstetrics, Paulista School of Medicine, Federal University of São Paulo (EPM-UNIFESP), São Paulo 04023-062, SP, Brazil; acarolrc@uol.com.br (A.C.R.C.); lunardozza@uol.com.br (L.M.M.N.); ldrossi@unifesp.br (L.D.R.); araujojred@terra.com.br (E.A.J.); 2 Department of Obstetrics and Gynecology, University of São Paulo (USP), São Paulo 01246-903, SP, Brazil; 3 Department of Obstetrics and Gynecology, Faculty of Medical Sciences of Santa Casa of São Paulo (FCMSCSP), São Paulo 01224-001, SP, Brazil; drgitedesco@gmail.com; 4 Albert Einstein Israelite College of Health Sciences, Albert Einstein Israelite Hospital, São Paulo 05653-000, SP, Brazil; gycallado@gmail.com; 5 Department of Gynecology and Obstetrics, Ribeirão Preto Medical School, University of São Paulo (FMRP-USP), Ribeirão Preto 14049-900, SP, Brazil; dralemar@uol.com.br

**Keywords:** fetal growth restriction, delivery, management, Doppler velocimetry, perinatal morbidity and mortality, fetal surveillance

## Abstract

Fetal growth restriction (FGR), a condition in which the fetus fails to achieve its growth and developmental potential, affects 5% to 10% of pregnancies and is associated with high rates of perinatal morbidity and mortality. There is currently insufficient high-quality evidence to define the optimal approach for diagnosing fetal growth restriction. In 2016, with the aim of standardizing clinical practice and enabling comparability across scientific studies, an expert opinion-based consensus was published. This document proposed unified terminology and clear diagnostic criteria for early- and late-onset fetal growth restriction (FGR). Because no effective treatment is available, careful assessment of fetal well-being and appropriate timing of delivery are the main tools for managing these fetuses. This decision should be based on gestational age and the severity of abnormalities identified on fetal surveillance tests, balancing the risks of prematurity against the risks of severe permanent sequelae or fetal death. The objective of this update is to analyze the most recent evidence on when and how to deliver pregnancies complicated by fetal growth restriction, emphasizing that specific abnormalities on fetal surveillance examinations warrant delivery at different gestational ages. To this end, a literature search of the PubMed/Medline and Latin America and the Caribbean Literature on Health Sciences (LILACS) databases was conducted using the terms fetal growth restriction, management, and delivery over the past ten years. Results were grouped into gestational age at delivery, mode of delivery, and methods of labor induction. The main fetal surveillance abnormalities prompting delivery in each gestational-age range were discussed, leading to the development of management flowcharts. Despite the lack of consensus in the literature and the limited number of randomized clinical trials guiding clinical decisions in FGR, the available evidence was summarized to assist clinicians in managing pregnancies complicated by FGR. It should be emphasized that there are few randomized clinical trials to guide management decisions in FGR.

## 1. Introduction

Fetal growth restriction (FGR) affects up to 10% of all births and compromises perinatal prognosis [1,2]. This condition represents one of the most critical risk factors for death in morphologically normal fetuses [3]. A study including 92,218 pregnancies demonstrated a fetal death rate of 9.7 per 1000 births when FGR was detected before delivery, compared with 18.9 per 1000 births when this complication was not diagnosed during prenatal care, highlighting the importance of appropriate management [3]. In addition, FGR is associated with neurological impairment in childhood and metabolic syndrome in adulthood [4,5]. The lower the birth weight percentile, the higher the perinatal morbidity and mortality [6].

To date, there is no gold-standard method for diagnosing FGR. In clinical practice, fetal weight is the parameter used to define whether a fetus has a growth deficit at a given gestational age (GA) [7]. The terms small for gestational age (SGA) and FGR are often used interchangeably. Many obstetric societies’ consensus statements agree on using a weight below the 10th percentile to define a fetus as small for gestational age [8]. The disadvantage of using this cutoff is the inclusion of a considerable proportion of constitutionally small fetuses with a lower risk of morbidity and mortality [8]. If the estimated fetal weight (EFW) or fetal abdominal circumference (AC) is below the 3rd percentile, diagnostic specificity for truly growth-restricted fetuses increases; however, this approach may fail to identify those with less severe forms of growth restriction who still have the potential for perinatal complications [9].

In cases of suspected growth restriction—defined as a fetus with an estimated weight below the 10th percentile—a rigorous evaluation of fetal anatomy, maternal serologic testing, and, in selected cases, fetal karyotype analysis (or microarray/exome analysis, when available) are of paramount importance to exclude congenital anomalies, infections, or genetic disorders as possible etiologies [8]. When these evaluations are within normal ranges, some authors suggest combining biometric parameters with functional assessments that reflect placental insufficiency, such as Doppler velocimetry of the uterine arteries (UtA), umbilical artery (UA), and fetal middle cerebral artery (MCA), as well as the presence of oligohydramnios or even biochemical markers, to more accurately identify truly growth-restricted fetuses requiring closer surveillance [8]. The PORTO Study demonstrated that abnormal UA Doppler findings and EFW below the 3rd percentile were the best and most consistent predictors of perinatal outcome [9]. Oligohydramnios was relevant when associated with weight at or below the 3rd percentile [9]. A secondary analysis of the same study showed that an abnormal cerebroplacental ratio (CPR) is associated with adverse perinatal outcomes in fetuses with an estimated weight below the 10th percentile [10]. A systematic review with meta-analysis concluded that incorporating CPR into the evaluation of fetuses with growth restriction may add value in predicting adverse perinatal outcomes [11].

Based on the relative predominance of biometric, placental, and fetal functional findings, two types of FGR are currently recognized, differing in their clinical presentation and perinatal morbidity and mortality [7,12]. Early-onset FGR is diagnosed before 32 weeks of gestation and is more frequently associated with preeclampsia and placental pathology; deterioration in UA Doppler velocimetry determines fetal compromise [12]. Hemodynamic redistribution and oligohydramnios are common findings in this form of FGR. With worsening hypoxemia, abnormalities in ductus venosus (DV) Doppler waveforms, as well as alterations in biophysical variables, may be observed, reflecting fetal acidosis [8].

Late-onset FGR manifests after 32 weeks of gestation and is less frequently associated with preeclampsia. In these cases, in which diffusion impairment may coexist with placental perfusion abnormalities, hemodynamic redistribution and abnormal CPR are common, often with normal UA Doppler findings. In these fetuses, diagnosis is more challenging, and tolerance to hypoxia is lower [8]. The 32-week cutoff differentiating early- from late-onset FGR was established by Savchev et al. [12], who demonstrated differences in clinical presentation and perinatal morbidity and mortality between the two forms.

Despite numerous publications, there is no consensus in the literature regarding the most appropriate criteria for defining FGR. To standardize clinical practice and allow comparability among scientific studies, an expert opinion–based consensus was published establishing unified nomenclature and clear diagnostic criteria for early- and late-onset FGR, as shown in Table 1 [13]. In 2020, a study validating these FGR criteria was published based on a secondary analysis of a prospective study. This study compared the FGR definition proposed in the 2016 consensus with the previous definition, which considered any fetus with an EFW below the 10th percentile as growth-restricted, regardless of Doppler criteria. The conclusion was that both definitions perform poorly in predicting adverse neonatal outcomes, although the 2016 definition was slightly superior [14]. Therefore, further robust studies are required to validate the parameters proposed in the 2016 expert consensus [15].

The major challenges in the management of FGR include accurate identification of fetuses at risk for adverse perinatal outcomes, prevention of fetal death (or severe permanent sequelae in the child), and determination of the optimal timing and mode of delivery [15]. As there is still no effective treatment to reverse or halt the progression of placental insufficiency, fetal surveillance and timely decision-making regarding delivery remain the cornerstone strategies in managing these pregnancies to achieve better outcomes [16]. It is important to note that randomized clinical trials guiding management in FGR are scarce and use heterogeneous criteria to define the condition [17]. Therefore, the objective of this review is to analyze the available evidence regarding when and how delivery should be performed in fetuses with growth restriction.

## 2. Methods

The present review builds on prior work that informed the development of national recommendations by the São Paulo Obstetrics and Gynecology Society (SOGESP). This review was conducted through an electronic search of the PubMed/MEDLINE and Latin American and Caribbean Health Sciences Literature (LILACS) databases, as well as international recommendations from major obstetric colleges and societies, to identify the most relevant articles addressing the timing and mode of delivery in FGR. Combinations of the following Medical Subject Headings (MeSH) terms were used: fetal growth restriction, delivery, and management.

The initial search was performed in May 2018, covering the period from January 2013 to May 2018, and was updated in April 2023, encompassing publications from May 2018 to April 2023. The search was limited to articles published in English and Portuguese.

The database search initially yielded 483 articles. After the update, the authors identified an additional 373 articles and four international recommendations from obstetric societies and colleges. After screening titles and abstracts, 63 articles from the initial search and 27 from the update remained. Of these, 58 and 23 articles, respectively, were read in full, and 42 and 15, respectively, were included in the final review. An additional 25 articles considered relevant to the topic but published outside the predefined search period were also included.

**Figure 1:**
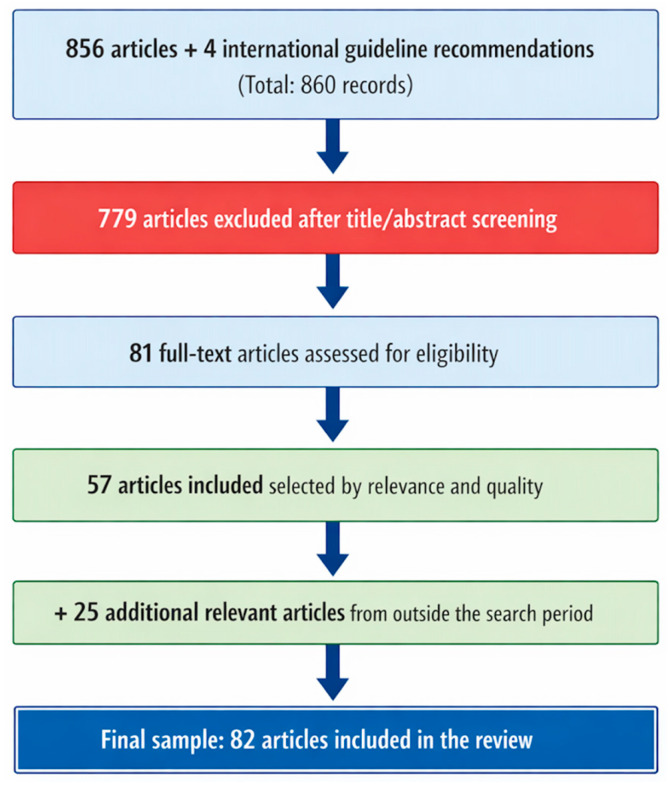
Literature search flow.

The study selection process is summarized in Figure 1. The authors prioritized articles with the greatest relevance to the topic, larger sample sizes, and higher levels of scientific evidence. Data extraction was initially performed by the lead reviewer and subsequently complemented by the other reviewers. The studies were categorized according to levels of scientific evidence (A, B, C, and D), as outlined below:

A. Experimental or observational studies of the highest consistency (meta-analyses or randomized clinical trials);

B. Experimental or observational studies of lower consistency (non-randomized clinical trials, observational studies, or case–control studies);

C. Case reports or case series (uncontrolled studies);

D. Opinion without critical appraisal, based on consensus statements, physiological studies, or animal models.

## 3. Results

Most available management protocols for FGR recommend combining multiple methods to assess fetal well-being in order to safely postpone delivery to more advanced gestational ages and reduce neonatal risks related to prematurity. The use of multiple assessment tools—such as fetal biometry, amniotic fluid volume measurement, cardiotocography, biophysical profile, and placental and fetal Doppler velocimetry—improves the prediction of fetal acidemia and fetal death compared with isolated tests [8]. The decision regarding delivery timing is based on gestational age (GA), the etiology of FGR, the degree of fetal compromise, the clinical team’s experience, and the technological resources available for fetal assessment and neonatal care. Ideally, management and delivery should be performed in a tertiary care center [7].

However, as with other aspects of FGR, there is no consensus in the literature regarding the optimal timing of delivery. Below, we review selected clinical trials that have attempted to clarify this issue.

### 3.1. Gestational Age at Delivery

A randomized clinical trial entitled The Growth Restriction Intervention Trial (GRIT) randomized pregnancies complicated by FGR between 24 and 36 weeks into two groups: immediate delivery (*n* = 296) or expectant management (*n* = 292). The groups were homogeneous with respect to maternal and fetal characteristics. In the expectant management group, delivery occurred on average 4.9 days after enrollment, and 40% of fetuses had absent (AEDF) or reversed end-diastolic flow (REDF) in the umbilical artery (UA). The immediate delivery group had fewer fetal deaths compared with the expectant management group (2% vs. 9%). Conversely, the combined rates of neonatal death and severe disability at two years of age were higher in the immediate delivery group (19% vs. 16%), although this difference was not statistically significant (OR 1.1; 0.7–1.8). However, when outcomes were analyzed in pregnancies at 31 weeks or less, these rates were significantly higher in the immediate delivery group (13% vs. 5%) [18].

Follow-up of the children between six and 13 years of age showed no differences between groups in cognition, language, motor development, or behavior [19]. These findings suggest that expectant management in extremely preterm growth-restricted fetuses, in the absence of a clear indication for delivery, results in more fetal deaths. In contrast, immediate delivery leads to a higher number of neonatal deaths. However, neither strategy improved long-term neurological prognosis [18,19].

As most studies addressing GA at delivery divide outcomes into deliveries before and after 34 weeks, this gestational-age-based division will be maintained for the analysis.

### 3.2. Delivery Before 34 Weeks

The TRUFFLE study (Trial of Randomized Umbilical and Fetal Flow in Europe) evaluated perinatal and long-term outcomes in fetuses with early-onset FGR. Pregnant women between 26 and 32 weeks with singleton pregnancies, fetal abdominal circumference (AC) below the 10th percentile, and UA pulsatility index (PI) above the 95th percentile were included. Participants were allocated to three groups according to the trigger for delivery:

(1) Reduced short-term variation on computerized cardiotocography (cCTG);
(2) Early abnormality in ductus venosus (DV) Doppler (PI > 95th percentile);
(3) Late DV abnormality (absent a-wave).

Most deliveries occurred for reasons other than those predefined in each group (maternal or other fetal indications), and only 32% were based on the assigned criteria. In the DV-based groups, cCTG served as a safety-net criterion, whereas no additional safety criterion was applied to the cCTG group.

Children in the cCTG group had lower survival without neurodevelopmental impairment at two years of age (77%) compared with those delivered based on DV abnormalities (83%); however, this difference was not statistically significant. Among survivors, children from the DV-monitoring groups had half the prevalence of neurological impairment compared with the cCTG group (7% vs. 15%, *p* = 0.049). The authors concluded that fetuses with early-onset FGR should be longitudinally monitored using DV Doppler and cCTG to determine the optimal timing of delivery [20].

A secondary analysis demonstrated that, at gestational ages below 32 weeks, it appears safe and beneficial for long-term outcomes to delay delivery until abnormalities are detected in DV PI, short-term variation on cCTG, or recurrent decelerations on conventional CTG [21]. Another study assessing outcomes at two years of age found better-than-expected prognoses, with particularly favorable neurological development among children delivered due to late DV abnormalities. No evidence was found to support delivery before 32 weeks based solely on fetal cerebral Doppler abnormalities [22].

Caradeux et al. [23] conducted a systematic review and meta-analysis evaluating the risk of death before 34 weeks in fetuses with absent or reversed end-diastolic flow in the UA and/or DV. Thirty-one studies were included, encompassing 5909 Doppler assessments. The odds ratios for fetal death were 3.59, 7.27, and 11.6 for growth-restricted fetuses with absent UA flow, reversed UA flow, and DV with absent or reversed a-wave, respectively.

The risk of fetal death associated with absent UA flow exceeded the risk of neonatal mortality or severe morbidity between 33 and 34 weeks. In cases of reversed UA flow and/or DV with absent or reversed a-wave, the risk of fetal death exceeded that of neonatal mortality and severe morbidity from 30 weeks onward. Only five studies separately analyzed DV Doppler, defining absent or reversed a-wave as severe; these studies showed that the risk of fetal death surpassed prematurity-related morbidity and mortality from 28 weeks onward [23].

Importantly, when FGR coexists with preeclampsia, both conditions reflect more severe placental dysfunction and may modify the expected trajectory of fetal compromise. Although this association is more frequent and clinically significant in early-onset disease, late-onset FGR may also be complicated by hypertensive disorders. In such cases, deterioration can occur more rapidly and unpredictably than in isolated FGR. Therefore, strict fetal surveillance and close integration of maternal and fetal assessment are warranted, as clinical worsening may necessitate earlier intervention [1].

### 3.3. Delivery After 34 Weeks

Evidence supporting delivery decisions in growth-restricted fetuses beyond 34 weeks can be drawn from The Disproportionate Intrauterine Growth Intervention Trial At Term (DIGITAT). This randomized clinical trial included 650 women with suspected FGR between 36 and 41 weeks and compared induction of labor (IOL) with expectant management at term.

Inclusion criteria were fetal weight and/or abdominal circumference (AC) below the 10th percentile and/or a decline in the fetal growth curve during the third trimester (based on the obstetrician’s subjective judgment). Doppler criteria were not included, and EFW/AC values below the 10th percentile were not stratified into lower percentiles; therefore, no distinction was made between truly growth-restricted fetuses and constitutionally small fetuses.

No differences were observed between the induction and expectant management groups regarding neonatal morbidity, cesarean delivery rates, or operative vaginal delivery rates. The authors concluded that expectant management with strict fetal surveillance may be adopted; however, induction of labor at term appears to be a reasonable approach [24].

In 2015, a study compared two management protocols for FGR defined as EFW below the 10th percentile. The second protocol, which individualized delivery timing according to umbilical artery (UA) Doppler findings, reduced rates of preterm birth and neonatal intensive care unit (NICU) admission compared with routine delivery at 37 weeks [25].

Figueras et al. [26] published a cohort study evaluating 509 pregnancies with small-for-gestational-age or growth-restricted fetuses, excluding those with absent or reversed UA flow. The best predictors of adverse perinatal outcome were cerebroplacental ratio (CPR) below the 10th percentile, mean uterine artery pulsatility index (PI) above the 95th percentile, and EFW below the 3rd percentile.

A systematic review published in 2015 comparing planned early delivery with expectant management in term pregnancies with suspected fetal compromise found insufficient evidence to support either strategy, highlighting the need for higher-quality studies with larger sample sizes [27].

Pilliod et al. [28] demonstrated that, from 38 weeks onward, the risk of perinatal death associated with expectant management for an additional week exceeded the risk associated with immediate delivery, particularly when birth weight was below the 3rd percentile. However, the study evaluated only birth weight and did not include Doppler or structured fetal surveillance data.

A United Kingdom study published in 2017 evaluated outcomes after implementing a risk-stratification protocol. Mean gestational age at delivery increased, while rates of adverse neonatal outcomes, NICU admission, induction of labor, and cesarean delivery decreased. No difference in perinatal mortality was observed, although the sample size was insufficient to adequately assess this outcome [29].

Subsequent studies comparing induction of labor with expectant management have shown mixed results. Some suggest that expectant management may reduce neonatal morbidity when Doppler findings are normal [30], whereas others indicate that induction at 37 weeks may reduce fetal death, particularly in late preterm pregnancies [31].

In March 2023, a prospective study evaluating risk-based management of late-onset FGR concluded that expectant management of low-risk fetuses below the 10th percentile is feasible and associated with fewer adverse perinatal outcomes, although randomized clinical trials are needed to confirm long-term safety [32].

Currently, two randomized clinical trials are underway to guide decision-making regarding the timing of delivery in late-onset FGR: TRUFFLE 2 and DRIGITAT. Both trials incorporate the umbilicocerebral ratio (UCR) as a key parameter, although most prior studies have used the CPR. Current evidence does not demonstrate clear superiority of one index over the other [33,34].

### 3.4. Practical Summary of Delivery Triggers by Gestational Age

Based on the evidence discussed above, Table 2 summarizes gestational age–specific delivery triggers in fetal growth restriction, integrating Doppler findings and markers of fetal deterioration to support clinical decision-making.

### 3.5. Mode of Delivery in FGR

It is important to emphasize that randomized clinical trials to support the choice of mode of delivery are lacking, and most protocols are based on expert opinion. Women with FGR are at increased risk of emergency cesarean delivery due to acute fetal distress during labor, even when UA Doppler findings are normal [35,36].

In a prospective cohort study, Cruz-Martínez et al. [36] compared 210 appropriate-for-gestational-age fetuses with 210 SGA fetuses with normal UA Doppler and found higher rates of cesarean delivery for fetal distress and neonatal acidemia among SGA fetuses compared with controls. Within the SGA group, pregnancies in which the fetus demonstrated brain-sparing (abnormal CPR) before induction had higher rates of cesarean delivery, cesarean delivery for fetal distress, and neonatal metabolic acidosis than those with normal MCA Doppler findings. The authors concluded that MCA Doppler assessment may help identify growth-restricted fetuses at higher risk of intrapartum asphyxia and adverse neonatal outcomes. These findings should be interpreted alongside other clinical factors—such as parity, cervical status, and institutional resources—when determining the most appropriate mode of delivery.

Bernardes et al. [37] compared outcomes from the HYPITAT and DIGITAT trials among women managed with induction of labor or expectant management with a Bishop score < 6. A total of 1172 women were included. No differences in cesarean delivery rates were observed between groups. Induction of labor in women with a Bishop score between 3 and 6 did not increase cesarean delivery rates or worsen neonatal outcomes. However, Doppler findings were not analyzed separately.

Retrospective studies have reported vaginal delivery rates around 60% in late-onset FGR undergoing induction, with higher cesarean rates associated with abnormal Doppler findings, Bishop score < 3, oxytocin use for induction, prematurity, and maternal morbidity [38,39,40].

In pregnancies with AEDF or REDF in the UA, most specialists recommend elective cesarean delivery [31]. Older studies report emergency cesarean rates ranging from 75% to 95% in such cases [41,42]. A prospective multicenter European study reported markedly higher perinatal mortality in cases of AEDF and REDF compared with preserved positive diastolic flow, supporting cesarean delivery in these situations [41]. Similarly, pregnancies with advanced fetal deterioration—such as DV abnormalities, abnormal BPP, or reduced short-term variation on cCTG—should generally be delivered by elective cesarean delivery [1,7,8,15,16].

In contrast, in cases of FGR with increased UA resistance but preserved diastolic flow, MCA vasodilation, or abnormal CPR, labor induction is feasible, provided that continuous and heightened intrapartum surveillance is ensured due to the increased risk of fetal asphyxia and emergency cesarean delivery [24,25,29,36,38,39].

### 3.6. Methods of Labor Induction in FGR

A Cochrane systematic review published in 2012 compared mechanical methods (intracervical balloon) with pharmacologic methods (misoprostol, prostaglandin E2, and/or oxytocin) for cervical ripening/IOL in patients without stratification by underlying pathology. The review pooled 9722 patients and concluded that mechanical and pharmacologic methods were associated with similar cesarean delivery rates. However, mechanical methods were associated with a lower risk of uterine hyperstimulation and fetal heart rate abnormalities. Compared with oxytocin, these methods were more effective for IOL [43]. Unfortunately, this review did not specify maternal conditions or FGR.

Few studies with small sample sizes have compared induction methods in pregnancies with SGA or growth-restricted fetuses. One clinical trial compared 46 women induced with 25 mcg misoprostol every 6 h (maximum of three doses) with 54 women undergoing intracervical balloon insertion. Pregnancies complicated by oligohydramnios (amniotic fluid index < 5) and AEDF/REDF in the UA were excluded. One patient in the misoprostol group developed tachysystole with CTG changes. Cesarean delivery rates were lower in the misoprostol group, with no significant differences in perinatal outcomes [44].

A retrospective cohort study compared the efficacy and safety of misoprostol (*n* = 20), dinoprostone (*n* = 21), and cervical balloon (*n* = 58) for IOL at term in pregnancies with fetuses with EFW below the 3rd percentile and normal Doppler findings. Pregnancies with oligohydramnios were excluded. The authors concluded that prostaglandins were more effective for IOL, as cesarean rates were lower, with no differences in tachysystole and/or adverse perinatal outcomes [45]. Another cohort study compared outcomes of IOL with misoprostol (*n* = 172), dinoprostone (*n* = 38), or cervical balloon plus oxytocin (*n* = 50) in pregnancies with SGA fetuses. There were no significant differences in vaginal delivery rates, cesarean delivery for acute fetal distress, or NICU admission. The study concluded that misoprostol was efficacious and safe in SGA fetuses, with efficacy and safety comparable to other IOL methods [46].

A systematic review and meta-analysis published in 2020 compared IOL methods in pregnancies with SGA fetuses and late-onset growth restriction. Results were stratified into three groups: (1) fetuses with EFW or AC < P10 regardless of Doppler; (2) late-onset FGR (EFW or AC < 3rd or between 3rd and 10th with abnormal cerebroplacental Doppler); and (3) SGA fetuses with EFW or AC between 3rd and 10th and normal Doppler. Data from 12 studies involving 1711 patients were analyzed. In the overall cohort, mechanical methods (balloon and Foley catheters) were associated with fewer unfavorable intrapartum outcomes, lower rates of cesarean delivery for fetal distress, fewer cases of tachysystole, and fewer CTG abnormalities. Direct comparisons between methods were not possible due to insufficient numbers and study heterogeneity. In the late-onset FGR subgroup, mechanical methods were also associated with fewer adverse effects than dinoprostone, with unfavorable intrapartum outcomes in 25.3% of the dinoprostone group versus 7.4% in the mechanical group, and cesarean delivery for fetal distress in 23.8% versus 6.2%, respectively. Comparison with misoprostol was not feasible. In the SGA subgroup, unfavorable intrapartum outcomes occurred in 8.4%, 18.6%, and 8.7% in the dinoprostone, misoprostol, and mechanical-method groups, respectively. The review concluded that evidence is limited; however, mechanical methods appear safer for IOL in SGA and late-onset growth-restricted fetuses [47]. Based on these data, the FIGO (International Federation of Gynecology and Obstetrics) protocol recommends prioritizing mechanical methods over prostaglandins for cervical ripening in cases of suspected or confirmed FGR. If a prostaglandin is chosen, a reversible option such as dinoprostone should be used [17].

In 2022, Iwai et al. [48] published a retrospective cohort study comparing an older IOL protocol with a more recent one. Under the earlier protocol, in fetuses with suspected growth restriction, oxytocin was maintained until delivery unless an indication arose to stop the drug; under the newer protocol, oxytocin is reduced or discontinued once the patient enters the active phase of labor. This protocol was associated with lower cesarean delivery rates, a lower incidence of tachysystole, and shorter second-stage duration, although these differences were not statistically significant. No differences were observed in other maternal or neonatal outcomes. The study suggests that in IOL for suspected FGR, oxytocin should be discontinued once the active phase of labor has reached [48].

### 3.7. International Guideline Recommendations

Over the past five years, several international guidelines from societies in Obstetrics and Gynecology and Ultrasound have been updated and/or published. In this recommendation, we summarize in Table 3 the most recent guidance from these societies regarding delivery in pregnancies complicated by FGR: FIGO [17], International Society of Ultrasound in Obstetrics and Gynecology (ISUOG) [49], Society for Maternal-Fetal Medicine (SMFM) [50], and American College of Obstetricians and Gynecologists (ACOG) [51].

## 4. Discussion

Although the available studies are not homogeneous with respect to the diagnostic and management criteria used—and therefore high-level, evidence-based recommendations to guide the optimal timing of delivery are lacking—several important considerations can be inferred from the available data. Developing protocols based on the best available evidence has the potential to reduce variability in clinical practice [1].

Within this context, the present review focuses on singleton pregnancies. The diagnosis, surveillance, and timing of delivery in twin gestations complicated by fetal growth restriction follow distinct criteria and management algorithms that vary according to chorionicity and specific complications (e.g., selective FGR in monochorionic twins). These conditions require dedicated consideration and were therefore beyond the scope of this review.

In the opinion of most authors, fetal surveillance should begin at 24–26 weeks (depending on institutional viability limits) and should include multiple modalities, such as Doppler velocimetry, computerized or conventional cardiotocography (cCTG/CTG), and biophysical profile (BPP) [16]. In addition, fetal death may occur unexpectedly in late-onset FGR, a risk that may be mitigated by intensifying fetal surveillance from 34 weeks onward [52].

Morbidity and mortality at the limits of viability must always be considered when deciding on delivery. Between 24 and 26 weeks, survival rates are below 50%, and among survivors, severe morbidity occurs in approximately 80% of cases. Neonatal survival increases by about 2% for each additional day of intrauterine life, exceeding 50% once 26 weeks are reached or when fetal weight reaches 500 g. In most centers, delivery at such early gestational ages is indicated only for maternal indications. Between 26 and 28 weeks, survival exceeds 50%, but survival without severe sequelae remains around 30%. Local viability data and parental preferences after appropriate counseling should always be considered [53,54].

Below, we discuss the role of individual surveillance parameters in FGR and their implications for delivery timing according to gestational age.

### 4.1. Doppler Velocimetry of the Uterine Arteries

Uterine artery Doppler is a noninvasive method that helps evaluate placental function in the maternal compartment [55]. It is one of the criteria that may support the diagnosis of FGR [13]. Some studies have shown that SGA fetuses with abnormal uterine artery (UtA) Doppler have approximately twice the risk of developing abnormal middle cerebral artery (MCA) Doppler findings before delivery, which may assist in planning fetal surveillance [56]. Some FGR management protocols include assessment of the mean UtA pulsatility index (PI) and recommend delivery at 37–38 weeks when it exceeds the 95th percentile [1]. However, longitudinal studies with serial uterine artery assessments have not demonstrated progressive worsening of Doppler findings from diagnosis to delivery; therefore, its value as a tool for ongoing fetal surveillance is questionable [55,57]. Although uterine artery Doppler may help predict adverse outcomes in FGR, it does not currently provide sufficient evidence to be considered an effective adjunct in determining delivery timing [58].

### 4.2. Doppler Velocimetry of the Umbilical Artery

Umbilical artery (UA) Doppler plays a central role in the diagnosis and prognostic assessment of fetuses with growth restriction. When UA PI is elevated but end-diastolic flow is preserved, the mean time to the development of additional fetal surveillance abnormalities is approximately two weeks. In cases of absent end-diastolic flow (AEDF), the mean time to cardiovascular deterioration is about five days, and in cases of reversed end-diastolic flow (REDF), approximately two days [17].

UA REDF is associated with adverse perinatal outcomes independently of gestational age at delivery, with reported sensitivity and specificity of approximately 60% [59]. From 30–32 weeks onward, in cases of UA REDF, the risk of fetal death exceeds prematurity-related risks, thereby justifying delivery [1,8,23]. In cases of UA AEDF, the risks of fetal death or severe compromise surpass prematurity-related risks around 34 weeks, and delivery should therefore be indicated from that point onward [1,8,23].

In both AEDF and REDF, due to the high risk of intrapartum fetal distress, the preferred mode of delivery is elective cesarean delivery [41].

When UA resistance is increased but diastolic flow remains positive, delivery should generally be considered no later than 37 weeks [24,25,31]. Some protocols recommend delivery from 34 weeks onward, particularly when strict fetal surveillance is not available [7,17,35,60]. In such cases, induction of labor may be considered; however, intrapartum fetal monitoring should be rigorous and continuous (CTG) [17,49].

Induction may be performed using mechanical or pharmacologic methods. Caution is warranted with misoprostol due to the risk of tachysystole and CTG abnormalities. For this reason, some authors and professional society protocols preferentially recommend mechanical methods for cervical ripening in FGR [1,17,47].

### 4.3. Doppler Velocimetry of the Middle Cerebral Artery

Middle cerebral artery (MCA) vasodilatation is considered a key manifestation of late-onset FGR and is valuable for both diagnosis and prediction of adverse outcomes [1,8,11,61]. A retrospective study demonstrated that, in growth-restricted fetuses beyond 34 weeks, the mean interval between MCA vasodilatation and fetal death was five days. For this reason, several protocols recommend fetal surveillance at least twice weekly in such cases [17,49]. Another recommendation is to repeat Doppler assessment within 24 h when MCA vasodilatation or an abnormal cerebroplacental ratio (CPR) is detected in order to reduce false-positive results [49].

Studies have shown an association between brain-sparing (MCA PI below the 5th percentile) and adverse perinatal and neurological outcomes; however, there is no evidence supporting delivery before term solely on this basis [36]. Current evidence suggests that delivery at 37 weeks may be appropriate in these cases [1,8,24,29,49]. Some protocols recommend delivery from 34 weeks onward, particularly when strict fetal surveillance is not available; however, prematurity-related risks must be carefully balanced against the risk of fetal death in such settings [7,17,35,60].

Delivery in growth-restricted fetuses with brain-sparing may be achieved through induction of labor using mechanical methods (e.g., cervical balloon) or pharmacologic agents (prostaglandins). Nevertheless, caution is warranted with misoprostol due to the risk of tachysystole and acute fetal distress [1,17,43,47]. In addition, intrapartum fetal monitoring must be strict and continuous (continuous CTG) [17,49].

### 4.4. Abnormal Cerebroplacental Ratio

The use of CPR in the evaluation of SGA fetuses increases sensitivity for identifying true FGR compared with assessment of UA and MCA separately [1]. A systematic review and meta-analysis published in 2018 concluded that CPR appears useful in predicting perinatal death in pregnancies with suspected FGR; however, randomized clinical trials are needed to determine whether incorporating CPR into management reduces perinatal mortality or adverse outcomes [62].

The umbilicocerebral ratio (UCR) is another metric derived from UA and MCA Doppler indices. Stampalija et al. [61] demonstrated in a prospective cohort study that abnormal UCR values (>1.5 at 32–33 weeks and >1.0 at 34–36 weeks) were associated with adverse perinatal outcomes.

There is no evidence supporting planned early delivery before term solely based on CPR abnormalities [36]. Similarly, there is no evidence supporting prolongation of pregnancy beyond term in such cases. Delivery at 37 weeks has therefore been suggested [24,26,29]. Some protocols recommend delivery from 34 weeks onward, especially when optimal fetal surveillance is unavailable [7,17,35,60]. As always, prematurity-related risks must be weighed against the risk of fetal compromise. Intrapartum management should follow the same principles described for fetuses with brain-sparing.

### 4.5. Doppler Velocimetry of the Ductus Venosus

Abnormal ductus venosus (DV) Doppler is considered the single most powerful parameter for predicting short-term fetal death in early-onset FGR [1]. An absent or reversed a-wave in the DV is associated with high perinatal mortality regardless of gestational age at delivery [63,64]. When this abnormality is present, delivery is recommended from 28 weeks onward, after administration of antenatal corticosteroids [64,65].

When DV PI exceeds the 95th percentile but the a-wave remains positive, the risk of perinatal mortality remains high (40–70%). In approximately 90% of cases, DV abnormalities occur 48–72 h before deterioration of the biophysical profile (BPP), and in 50% of cases before worsening short-term variation (STV) on computerized CTG (cCTG). In this scenario, the preserved a-wave provides an opportunity to administer corticosteroids before delivery [1,8,20]. Given the high risk of fetal compromise, elective cesarean delivery is recommended in cases of DV abnormalities [41].

**Figure 2:**
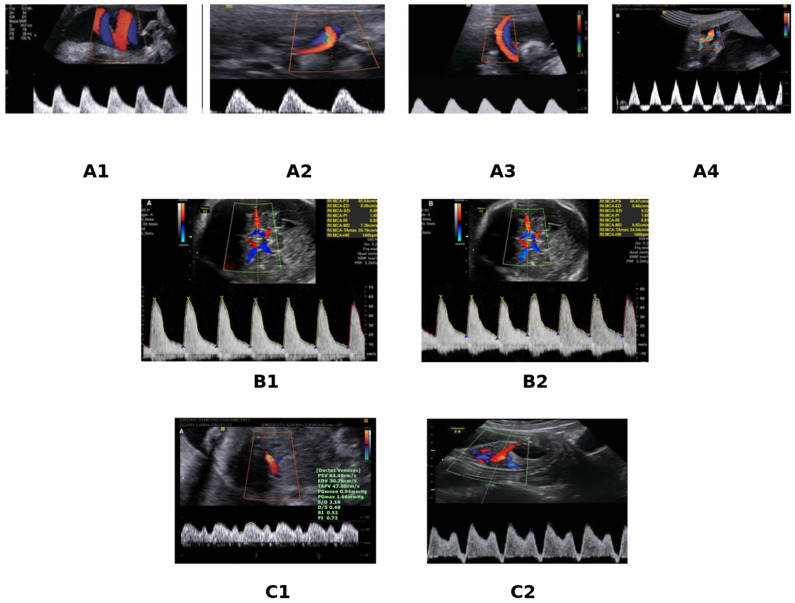
Progressive Doppler changes in fetal growth restriction reflecting placental insufficiency and fetal hemodynamic adaptation. (**A**) Umbilical artery Doppler: progressive increase in placental vascular resistance, from normal forward end-diastolic flow (**A1**) to increased placental resistance with reduced diastolic flow (**A2**), absent end-diastolic flow (AEDF) (**A3**), and reversed end-diastolic flow (REDF) (**A4**). (**B**) Middle cerebral artery: normal waveform (**B1**) compared with reduced pulsatility index indicating cerebral vasodilatation and the brain-sparing effect (**B2**). (**C**) Ductus venosus: normal waveform with preserved a-wave (**C1**) versus abnormal pattern with reversed a-wave (**C2**), reflecting advanced fetal hemodynamic deterioration and impending cardiac decompensation.

Taken together, these Doppler parameters reflect the well-established sequence of fetal hemodynamic deterioration in placental insufficiency: initial increase in umbilical artery resistance, followed by cerebral redistribution, and finally ductus venosus abnormalities. This progressive pattern is illustrated in Figure 2.

### 4.6. Biophysical Profile

Observational studies have demonstrated a correlation between abnormal BPP and perinatal mortality [66]. The parameters most strongly associated with fetal acidemia are absent fetal tone and absent gross body movements. A BPP score ≤ 4 is associated with umbilical artery pH < 7.20 [66].

Recent studies in early-onset FGR and very preterm fetuses suggest that BPP alone is not reliable for predicting fetal well-being over the subsequent 24 h. An integrated approach combining Doppler velocimetry and cCTG has therefore been recommended [67]. A Cochrane meta-analysis by Alfirevic and Neilson [68] found no evidence that BPP alone improves outcomes in high-risk pregnancies. Moreover, BPP has limited utility in predicting impending fetal deterioration, which is more accurately detected by Doppler changes [16].

Nevertheless, some authors and professional societies recommend BPP when cCTG is unavailable [69]. This recommendation is extrapolated from TRUFFLE data showing that combining multiple surveillance modalities improves outcomes; however, it should be emphasized that TRUFFLE used Doppler and cCTG—not BPP—for early-onset FGR assessment [20].

A BPP ≤ 4 indicates delivery by elective cesarean from viability onward [8,17,49,66]. A BPP score of 6 that persists after repeat evaluation at six hours also warrants delivery when gestational age exceeds 34 weeks [16].

### 4.7. Cardiotocography

Conventional CTG, due to its high sensitivity, yields high false-positive rates in predicting adverse perinatal outcomes. One major limitation is subjective interpretation, which becomes especially challenging in very preterm and physiologically immature fetuses [1]. A Brazilian study concluded that late decelerations, more ominous variable decelerations, and prolonged bradycardia in fetuses with abnormal UA Doppler were predictors of acidemia [70]. A Cochrane meta-analysis published in 2000 did not demonstrate that antepartum CTG in high-risk pregnancies reduced perinatal mortality [71]. However, tracings with absent variability and/or spontaneous decelerations occur late in the follow-up of compromised fetuses and may precede fetal death; thus, they represent indications for delivery [1,17,49,72].

Computerized CTG (cCTG) represented an advance in fetal surveillance in growth restriction because it allows assessment of STV [73]. Studies indicate that STV has sufficient sensitivity to detect fetal deterioration and has a predictive value like DV Doppler for short-term fetal mortality. STV deterioration occurs before DV abnormalities in 50% of cases [74]. An STV < 3.5 milliseconds has excellent accuracy for acidemia and UA pH < 7.20 at birth [8]. Another advantage of cCTG is that the assessment is less subjective than conventional CTG interpretation.

TRUFFLE demonstrated that combining DV Doppler with cCTG in decisions regarding delivery resulted in better perinatal outcomes [21]. Other studies reported similar results [75]. STV < 3.5 ms indicates the need for delivery at viability by elective cesarean due to the high risk of intrapartum fetal compromise and perinatal death [1,8,74].

Computerized CTG is less frequently discussed in the evaluation of late-onset FGR. In 2021, a prospective cohort study reported lower STV values in fetuses with late-onset growth restriction compared with appropriately grown fetuses. Additionally, lower STV was observed among growth-restricted neonates admitted to the NICU compared with those without complications [76]. However, cCTG is not available in all settings.

### 4.8. Amniotic Fluid Assessment

A meta-analysis including 18 clinical trials found an association between reduced amniotic fluid volume (amniotic fluid index [AFI] < 50 mm) and a 5 min Apgar score < 7, as well as a higher incidence of intrapartum fetal distress, without an association with acidosis or perinatal death [77]. Longitudinal studies in women with early-onset FGR have shown progressive reduction in amniotic fluid volume, and one week before acute deterioration, oligohydramnios is present in 20% to 30% of cases [1]. Many services consider an AFI < 50 mm or a deepest vertical pocket < 20 mm as an indication for delivery from 37 weeks onward. In such cases, IOL is feasible with strict fetal monitoring. However, to date, the inclusion of oligohydramnios in FGR management protocols cannot be supported by the published literature, and more studies are needed to validate its use [1]. The FIGO protocol includes oligohydramnios as one criterion for delivery but emphasizes that available studies have not demonstrated an association with acidosis and consequent severe injury or fetal death [17].

### 4.9. Low-Weight Fetuses with Normal Surveillance Tests

Small-for-gestational-age (SGA) fetuses are those with estimated weight between the 3rd and 10th percentiles and normal Doppler findings. The prognosis of these children is generally similar to that of appropriately grown fetuses. Recent recommendations have suggested delivery between 38 and 39 weeks in these cases, considering that the leading cause of perinatal death at term is stillbirth and that some fetuses classified as SGA may have some degree of compromise not detectable by available biophysical methods [17,49,51]. These recommendations are mainly based on DIGITAT data to support delivery at 38–39 weeks [24]. However, it should be remembered that DIGITAT included patients with EFW < 10th percentile and did not apply Doppler criteria or stratify fetuses into specific weight groups below the 10th percentile.

In 2023, a prospective study used biometric, growth-curve, and Doppler criteria to distinguish between SGA and growth-restricted fetuses. In that study, fetuses classified as SGA (low risk) were followed until 41 weeks with favorable outcomes and good perinatal results. The authors concluded that appropriately distinguishing SGA from growth-restricted fetuses may be advantageous for delivery timing, avoiding unnecessary interventions in the low-risk group [32]. However, randomized clinical trials are needed to recommend the optimal gestational age at delivery for SGA fetuses with higher levels of evidence [32].

The most recent recommendations indicate that delivery of fetuses considered SGA may be performed between 38 and 39 weeks, depending on the availability of fetal surveillance methods [1,8,17,29,49,51]. Induction of labor may be used and can be performed using mechanical or pharmacologic methods, with caution regarding misoprostol due to the risk of uterine tachysystole and intrapartum fetal compromise [43,47]. Conversely, studies have not identified benefits of prolonging pregnancy beyond 37–38 weeks when fetuses have EFW and/or AC below the 3rd percentile, even with normal Doppler findings [17,24,28,49,51]. From that gestational age onward, IOL is recommended by most authors and should follow the same criteria described above, with preference for mechanical methods and strict intrapartum fetal surveillance (continuous cardiotocography) [17,43,47].

In addition to the evidence summarized above, recent retrospective data have explored predictors of successful induction in pregnancies complicated by FGR, suggesting that induction at ≥37 weeks and the absence of significant Doppler abnormalities may be associated with higher rates of vaginal delivery. These findings further support individualized planning of induction and mode of delivery in SGA and late-onset FGR [78].

An emerging approach to refining risk stratification before induction of labor involves the clinical application of the Fetal Medicine Foundation (FMF) 36-week preeclampsia risk model. Although originally developed to predict term preeclampsia, recent evidence suggests that higher estimated risk using the FMF algorithm is associated with an increased likelihood of intrapartum fetal compromise requiring cesarean delivery following induction of labor. Importantly, fetal growth restriction represents a major contributing condition among affected cases. This model may therefore provide an additional tool to identify pregnancies at higher risk of intrapartum compromise and assist in counseling and individualized planning of delivery, particularly when induction is considered in growth-restricted or borderline cases [79].

The distinction between small-for-gestational-age (SGA) and fetal growth restriction (FGR) is clinically relevant, as it directly influences surveillance strategies and timing of delivery. Table 4 summarizes the key diagnostic and management differences between these two conditions.

Although SGA fetuses with normal Doppler findings generally have favorable outcomes, an important clinical challenge arises when fetuses fall outside strict diagnostic criteria for fetal growth restriction but exhibit functional signs of placental insufficiency. This includes cases such as fetuses with estimated fetal weight just above the 10th percentile accompanied by abnormal Doppler findings, particularly an altered cerebroplacental ratio.

While these fetuses do not formally meet the Delphi consensus criteria for FGR, accumulating evidence suggests that abnormal Doppler parameters reflect impaired placental function and increased perinatal risk, even when biometric thresholds are not crossed. In clinical practice, such cases should not be managed as constitutionally small fetuses. Instead, closer fetal surveillance is warranted, and delivery timing should be individualized according to gestational age, Doppler progression, and the availability of fetal monitoring resources. This pragmatic approach acknowledges the limitations of purely biometric definitions and supports risk-based management rather than rigid categorization. From a practical standpoint, fetuses presenting abnormal functional parameters despite borderline biometric measurements are generally followed according to growth-restricted surveillance protocols, given their higher risk profile [11,13,17,49].

Table 5 summarizes the frequency of fetal surveillance in SGA and growth-restricted fetuses.

### 4.10. Antenatal Corticosteroids and Magnesium Sulfate to Reduce Adverse Neonatal Outcomes in FGR

Corticosteroids should be administered between 24 and 34 weeks, preferably as a single course and as close to delivery as possible (within the preceding 7 days at most), to accelerate pulmonary maturation and reduce the risk of parenchymal hemorrhages, particularly in the central nervous system [80]. Two antenatal corticosteroid regimens may be used: 12 mg intramuscular betamethasone given 24 h apart (two doses), or 6 mg dexamethasone every 12 h for two days (four doses) [81].

According to the ACOG, a single repeat course of corticosteroids may be considered once before 34 weeks if the first course was administered more than 7 days earlier and the clinician judges that delivery is likely within the next 7 days [81]. The effects of this second course have not been evaluated explicitly in growth-restricted fetuses [17]. Due to potential maternal and fetal adverse effects, more than two courses of corticosteroids are no longer recommended [82].

In 2016, Saccone and Berghella [81] conducted a systematic review and meta-analysis of corticosteroid effects on FGR. The authors demonstrated a benefit of corticosteroids in reducing neonatal respiratory distress when administered between 34 and 36 6/7 weeks in women at immediate risk of late preterm birth who had not received a prior course [80]. However, no studies were explicitly performed in the FGR population. SMFM and ACOG recommend this approach in cases of FGR [50,51].

It is important to note that immediately after corticosteroid administration, Doppler indices may appear improved; however, this finding is transient [82]. In cases of FGR with severe Doppler abnormalities, corticosteroids should preferably be administered with the patient hospitalized to allow rigorous fetal monitoring. Corticosteroids may cause fluid retention and increased fetal circulating volume, which can threaten the survival of a growth-restricted fetus with impaired cardiac function. In this context, the absence of a transient improvement in UA AEDF following corticosteroid administration may be a predictor of fetal deterioration [17].

**Figure 3:**
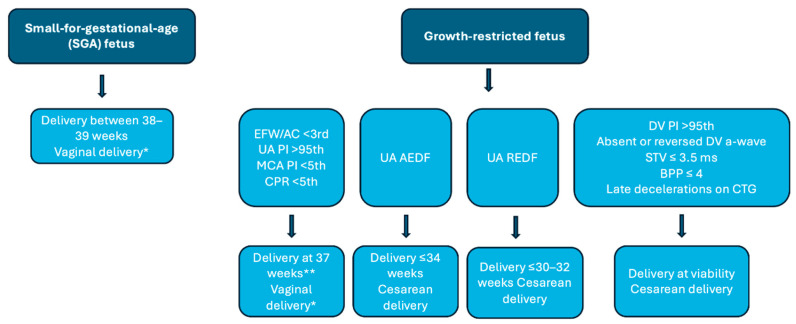
Management in cases of small-for-gestational-age and growth-restricted fetuses. * Strict intrapartum fetal surveillance is required in cases with abnormal Doppler findings, with continuous cardiotocography. Caution is advised with the use of prostaglandins; mechanical methods are preferred. ** In cases of FGR with abnormal Doppler findings, delivery at 37 weeks is recommended if strict fetal surveillance is available at the institution. Antenatal corticosteroids are indicated for deliveries before 34 weeks. Neuroprotection with magnesium sulfate is indicated for deliveries before 32 weeks.

For deliveries before 32 weeks, magnesium sulfate is recommended for neuroprotection, as in any preterm fetus at imminent risk of birth. Maternal dose and administration are the same as those used for magnesium sulfate in preeclampsia with severe features [83]. Studies evaluating neonatal outcomes with corticosteroids and magnesium sulfate in specific groups of pregnant women have shown benefits of these interventions in growth-restricted neonates [84]. Figure 3 presents the management protocol based on the evidence summarized in this review.

## 5. Final Recommendations

In cases of FGR, detection of an absent or reversed DV a-wave, reduced STV (<3.5 ms) on cCTG, and/or recurrent late decelerations on conventional CTG are indications for delivery. In the presence of AEDF in the UAs, delivery may be indicated from 34 weeks onward, supported by multiple fetal surveillance parameters. When REDF is present in the UAs, delivery may be indicated from 30 weeks onward, likewise supported by a multiparametric fetal assessment. Administration of a single course of maternal betamethasone before deliveries at gestational ages below 34 weeks is essential, provided that multiple fetal surveillance parameters allow postponement of delivery for at least 24 h. Perinatal neuroprotection with magnesium sulfate should be administered immediately before delivery in growth-restricted fetuses at GAs below 32 weeks. (A) In FGR with increased UA PI (with preserved diastolic flow), persistent MCA abnormality, or abnormal CPR, delivery is recommended by 37 weeks, provided that strict fetal surveillance is available until that time. For SGA fetuses with EFW, the 3rd and 10th percentiles and normal Doppler findings, delivery is recommended between 38 and 39 weeks, depending on the resources available to maintain fetal surveillance, and preferably via the vaginal route, with rigorous intrapartum fetal monitoring. FGR alone is not an indication for cesarean delivery. Cesarean delivery is recommended in cases of FGR with AEDF or REDF in the UA, abnormal DV Doppler, reduced STV on cCTG, recurrent late decelerations on conventional CTG, and a BPP score ≤ 4. In FGR, induction of labor may be performed using mechanical or pharmacologic methods even when UA Doppler is abnormal (with preserved diastolic flow), and/or when MCA Doppler or CPR is abnormal. However, the risk of intrapartum fetal asphyxia is higher than in uncomplicated pregnancies; therefore, continuous surveillance with conventional CTG is recommended. For cervical ripening before IOL in growth-restricted fetuses, mechanical methods should be preferred. If prostaglandins are used, a reversible agent such as dinoprostone should be the option.

## 6. Conclusions

Despite the relevance of this topic to obstetric practice, there is no consensus on the timing and mode of delivery in FGR. However, numerous studies have sought to standardize management and develop efficient, consistent protocols to improve perinatal outcomes. Thus, drawing on the breadth of the available literature, it is possible to formulate recommendations to guide clinical care. Nonetheless, it must be emphasized that, in more complex cases, tertiary-level support and a robust referral system are required to meet perinatal needs; such referral services must be accessible and available.

Because there is no effective treatment to reverse FGR, it is essential—within the limits of diagnostic performance—to establish criteria for timely pregnancy interruption across a broad spectrum of disease severity. The risks of fetal death and of neonatal morbidity and mortality due to placental insufficiency, as well as those attributable to prematurity, must be central to this decision-making process. In this framework, gestational age, the etiology of FGR, its classification, assessment of placental function (Doppler velocimetry), and evaluation of fetal biometry and well-being (biophysical and hemodynamic parameters) are necessarily the key domains to be addressed for appropriate clinical management. Inadequate assessment may lead to irreversible harm, underscoring the need to define the optimal timing of birth based on careful interpretation of the available data.

In early-onset FGR, it is crucial to emphasize the role of umbilical artery Doppler findings—which reflect placental function and the degree of compromise (AEDF or REDF)—as well as DV Doppler and CTG, especially cCTG, in determining delivery indications for fetuses at GAs below 34 weeks. In late-onset FGR, abnormalities on UA Doppler (which often show preserved AEDF), MCA Doppler, or an abnormal cerebroplacental ratio may support pregnancy interruption at term or upon pulmonary maturity, depending on the availability and capability of fetal surveillance methods at each institution.

Future perspectives in the management of late-onset fetal growth restriction continue to evolve. Two randomized clinical trials are currently underway with the objective of refining decision-making regarding the timing of delivery in this population: TRUFFLE-2 and DIGITAT. These studies aim to evaluate whether delivery strategies guided by fetal surveillance parameters can optimize perinatal and long-term outcomes while minimizing the risks associated with iatrogenic prematurity.

Together, TRUFFLE-2 and DIGITAT are expected to provide higher-quality evidence to support individualized management of late-onset FGR, helping clinicians balance the competing risks of continued intrauterine exposure to placental insufficiency against those related to preterm delivery [33,34].

Beyond the immediate perinatal period, fetal growth restriction has been associated with long-term consequences extending into childhood and adulthood. Neurodevelopmental impairment remains a concern, particularly in early-onset disease, and antepartum surveillance parameters have been correlated with later infant outcomes [4]. Although many survivors demonstrate reassuring global cognitive performance, subtle deficits in executive and motor function have been reported in some cohorts. Furthermore, the concept of fetal programming supports the association between impaired intrauterine growth and increased risks of hypertension, insulin resistance, and cardiovascular disease in adulthood [5,7]. These long-term implications reinforce the importance of accurate risk stratification, appropriate delivery planning, and structured postnatal follow-up.

Based on the available scientific evidence, the following conclusions can be drawn. In pregnant women at high risk for placental insufficiency, UA Doppler assessment reduces perinatal morbidity and mortality and is indispensable in pregnancies complicated by FGR. Doppler of the ductus venosus (DV) predicts acidemia and adverse neonatal outcomes and should be performed in preterm pregnancies with abnormal UA Doppler and/or evidence of hemodynamic redistribution. Computerized CTG (cCTG) with STV analysis is sensitive for detecting acute fetal compromise in the short term. In late-onset FGR, MCA Doppler and an abnormal CPR are associated with adverse perinatal outcomes and should be considered when planning delivery timing. This review, comprising the principal and most recent evidence on FGR, is intended to support the present proposal of summarizing recommendations that may assist professionals caring for pregnancies complicated by FGR.

## Figures and Tables

**Table 1:** Diagnostic criteria for fetal growth restriction based on expert consensus (Delphi method).

Early-Onset Fetal Growth Restriction (<32 Weeks)	Late-Onset Fetal Growth Restriction (≥32 Weeks)
AC/EFW < 3rd or umbilical artery (UA) Doppler with absent end-diastolic flow	AC/EFW < 3rd or at least two of the following three criteria:
OR	• AC/EFW < 10th
AC/EFW < 10th plus uterine artery (UtA) pulsatility index (PI) > 95th or UA PI > 95th	• AC/EFW drop of more than two quartiles on the fetal growth curve
	• Cerebroplacental ratio (CPR) < 5th or UA PI > 95th
Structural fetal malformations excluded	Structural fetal malformations excluded

**Table 2:** Gestational Age-Specific Indications for Delivery in Fetal Growth Restriction.

Gestational Age	Indication for Delivery
At fetal viability	Growth-restricted fetus with absent or reversed ductus venosus a-wave, biophysical profile (BPP) ≤ 4 or recurrent late/prolonged decelerations on CTG
30–32 weeks	Persistent reversed end-diastolic flow (REDF) in the umbilical artery
34 weeks	Persistent absent end-diastolic flow (AEDF) in the umbilical artery
37 weeks	FGR with normal fetal Doppler or mild fetal Doppler abnormalities (umbilical artery PI > 95th percentile, middle cerebral artery PI < 5th percentile, or cerebroplacental ratio < 5th percentile)

**Table 3:** Delivery in Fetal Growth Restriction According to International Society Guidelines.

	FIGO (2021)	ISUOG (2020)	SMFM (2020)/ACOG (2021)
FGR diagnosis	Delphi expert consensus (2016)	Delphi expert consensus (2016)	EFW and/or AC < 10th
EFW between 3rd and 10th with normal Doppler	Delivery between 37–39 weeks; mode of delivery ^d^	Delivery between 38–39 weeks; mode of delivery ^d^	Delivery between 38–39 weeks
EFW < 3rd with normal Doppler	Delivery between 36–38 weeks; mode of delivery ^d^	Delivery between 36–37 6/7 weeks; mode of delivery ^d^	Delivery at 37 weeks
Early Doppler abnormalities (UA PI > 95th, MCA < 5th, CPR < 5th; UtA PI > 95th)	Delivery between 34–37 weeks ^a^; labor induction ^d^ or cesarean section	Delivery between 36–37 6/7 weeks if UA PI > 95th ^b^; mode of delivery ^d^	Delivery at 37 weeks
UA absent end-diastolic flow	Delivery between 32–34 weeks; cesarean section	Delivery at 34 weeks; cesarean section	Delivery between 33–34 weeks; cesarean section
UA reversed end-diastolic flow	Delivery between 30–32 weeks; cesarean section	Delivery between 32–33 6/7 weeks; cesarean section	Delivery between 30–32 weeks; cesarean section
DV abnormality (PI > 95th, absent or reversed a-wave)	Delivery between 26–30 weeks; DV absent/reversed a-wave—26 weeks; cesarean section	Absent/reversed a-wave: delivery between 26 and 31 6/7 weeks; cesarean section	Does not recommend routine assessment ^c^
STV	STV < 3.0 ms—26 weeks; STV < 3.5 ms—30 weeks; STV < 4.5 ms—32 weeks; cesarean section	STV < 2.6 ms—26–28 6/7 weeks; STV < 3.0 ms—29–31 6/7 weeks; STV < 3.5 ms—32–33 6/7 weeks; STV < 4.5 ms—34 weeks; cesarean section	Does not recommend routine assessment
Absolute indication for delivery from viability	Maternal; CTG abnormalities (sinusoidal pattern, recurrent decelerations, absent variability with recurrent decelerations); BPP 0 or 2 (30 min) or 4 (60 min); STV < 2.6 ms; cesarean	Maternal; late decelerations on CTG; BPP ≤ 4; cesarean section	Late decelerations on CTG; cesarean section
Antenatal corticosteroids	If delivery < 34 weeks	If delivery < 34 weeks	If delivery < 34 weeks or 34–36 6/7 weeks if no prior course and no contraindications
Magnesium sulfate	If delivery < 32 weeks	Up to GA recommended by local guideline	If delivery < 32 weeks

^a^—FIGO includes oligohydramnios and suboptimal fetal growth in this management category; ^b^—ISUOG considers only UA PI > 95th in this category; in late-onset FGR, it recommends delivery between 38 and 39 weeks if there are signs of cerebral redistribution; ^c^—SMFM and ACOG do not recommend routine assessment of UtA, MCA, and DV Doppler in the management of early- or late-onset FGR; ^d^—FIGO and ISUOG recommend continuous intrapartum monitoring during labor for fetuses with growth restriction.

**Table 4:** Key diagnostic and management differences between small-for-gestational-age (SGA) and fetal growth restriction (FGR).

Parameter	Small-for-Gestational-Age (SGA)	Fetal Growth Restriction (FGR)
Definition	EFW between the 3rd and 10th percentile with normal Doppler findings	Diagnosed according to Delphi consensus criteria (2016), incorporating gestational-age–specific biometric and Doppler abnormalities
Placental function	Usually preserved	Impaired (placental insufficiency)
Doppler findings	Normal UA, MCA, CPR, DV	May show abnormal UA, MCA, CPR, DV or progressive hemodynamic deterioration
Risk of adverse outcome	Generally low	Increased risk of stillbirth and neonatal morbidity
Surveillance	Weekly or biweekly depending on GA	At least weekly; more frequent or inpatient monitoring if Doppler abnormalities are present
Timing of delivery	38–39 weeks in most cases	Based on gestational age and severity of Doppler abnormalities

**Table 5:** Frequency of fetal surveillance in small-for-gestational-age and growth-restricted fetuses.

Clinical Condition	Recommended Fetal Surveillance Frequency
Small for gestational age (SGA) fetus	Fetal surveillance * every two weeks until 37 weeks and weekly from 37 weeks until delivery
Growth-restricted fetus with normal fetal Doppler (EFW/AC < 3rd or between 3rd and 10th percentile with abnormal uterine artery Doppler only)	Weekly fetal surveillance until delivery
Growth-restricted fetus with abnormal umbilical artery Doppler (with positive diastolic flow), middle cerebral artery Doppler, or cerebroplacental ratio	Fetal surveillance 2 to 3 times per week until delivery
Growth-restricted fetus with absent end-diastolic flow on umbilical artery Doppler	Hospitalization and daily fetal surveillance until delivery
Growth-restricted fetus with reversed end-diastolic flow on umbilical artery Doppler	Hospitalization and daily fetal surveillance until delivery
Growth-restricted fetus with ductus venosus PI > 95th percentile	Hospitalization and daily or twice-daily fetal surveillance until delivery (while awaiting completion of antenatal corticosteroids)
Growth-restricted fetus with absent or reversed ductus venosus a-wave, BPP ≤ 4, STV ≤ 3.5 ms, or recurrent decelerations on CTG	Hospitalization and delivery at viability

* The frequency and type of fetal surveillance depend on the methods available at each institution; however, Doppler assessment is indispensable in the follow-up of growth-restricted fetuses. Doppler evaluation may be combined with biophysical profile or conventional or computerized cardiotocography.

## Data Availability

The data presented in this study are available on request from the corresponding author.
